# Biocompatible Magnetic Fluids of Co-Doped Iron Oxide Nanoparticles with Tunable Magnetic Properties

**DOI:** 10.3390/nano10061019

**Published:** 2020-05-27

**Authors:** Silvio Dutz, Norbert Buske, Joachim Landers, Christine Gräfe, Heiko Wende, Joachim H. Clement

**Affiliations:** 1 Institute of Biomedical Engineering and Informatics (BMTI), Technische Universität Ilmenau, D-98693 Ilmenau, Germany; 2 Department of Nano Biophotonics, Leibniz Institute of Photonic Technology (IPHT), D-07745 Jena, Germany; 3 MagneticFluids, Köpenicker Landstraße 203, D-12437 Berlin, Germany; ngb.buske@gmail.com; 4 Faculty of Physics and Center for Nanointegration Duisburg-Essen (CENIDE), University of Duisburg-Essen, D-47057 Duisburg, Germany; joachim.landers@uni-due.de (J.L.); heiko.wende@uni-due.de (H.W.); 5 Department Hematology and Oncology, Jena University Hospital, D-07747 Jena, Germany; christine.graefe@med.uni-jena.de (C.G.); joachim.clement@med.uni-jena.de (J.H.C.)

**Keywords:** cobalt ferrite, coercivity, ferrimagnetism, magnetic fluid hyperthermia, magnetic nanoparticles, magnetite

## Abstract

Magnetite (Fe_3_O_4_) particles with a diameter around 10 nm have a very low coercivity (H_c_) and relative remnant magnetization (M_r_/M_s_), which is unfavorable for magnetic fluid hyperthermia. In contrast, cobalt ferrite (CoFe_2_O_4_) particles of the same size have a very high H_c_ and M_r_/M_s_, which is magnetically too hard to obtain suitable specific heating power (SHP) in hyperthermia. For the optimization of the magnetic properties, the Fe^2+^ ions of magnetite were substituted by Co^2+^ step by step, which results in a Co doped iron oxide inverse spinel with an adjustable Fe^2+^ substitution degree in the full range of pure iron oxide up to pure cobalt ferrite. The obtained magnetic nanoparticles were characterized regarding their structural and magnetic properties as well as their cell toxicity. The pure iron oxide particles showed an average size of 8 nm, which increased up to 12 nm for the cobalt ferrite. For ferrofluids containing the prepared particles, only a limited dependence of H_c_ and M_r_/M_s_ on the Co content in the particles was found, which confirms a stable dispersion of the particles within the ferrofluid. For dry particles, a strong correlation between the Co content and the resulting H_c_ and M_r_/M_s_ was detected. For small substitution degrees, only a slight increase in H_c_ was found for the increasing Co content, whereas for a substitution of more than 10% of the Fe atoms by Co, a strong linear increase in H_c_ and M_r_/M_s_ was obtained. Mössbauer spectroscopy revealed predominantly Fe^3+^ in all samples, while also verifying an ordered magnetic structure with a low to moderate surface spin canting. Relative spectral areas of Mössbauer subspectra indicated a mainly random distribution of Co^2+^ ions rather than the more pronounced octahedral site-preference of bulk CoFe_2_O_4_. Cell vitality studies confirmed no increased toxicity of the Co-doped iron oxide nanoparticles compared to the pure iron oxide ones. Magnetic heating performance was confirmed to be a function of coercivity as well. The here presented non-toxic magnetic nanoparticle system enables the tuning of the magnetic properties of the particles without a remarkable change in particles size. The found heating performance is suitable for magnetic hyperthermia application.

## 1. Introduction

Magnetic nanoparticles (MNPs) and their biocompatible suspensions (ferrofluids) are very promising materials for biomedical applications [1,2]. Because of their small size and their high surface-to-volume ratio, MNPs show properties which differ from those of the bulk material. Due to their magnetic behavior, MNPs enable a mechanical manipulation in an external field or field gradient, a magnetic determination of their location, and a heating of the particles by an external alternating magnetic field, so that they cannot only be used in therapy, but also in diagnostics and as tracer materials. A broad range of applications like in hyperthermia [3,4], in drug targeting [5] or as contrast and tracer agent for medical imaging like magnetic resonance imaging (MRI) [6] or magnetic particle imaging (MPI) [7,8] of the MNPs are described in the literature.

A lot of different MNP systems with specific cores and coatings have been developed and tested for medical application. Because of their high biocompatibility, iron oxide MNPs are of particular interest for application in medicine [9,10]. Due to a good stability against agglomeration and sedimentation as well as a high cellular uptake, a lot of research is focused on the application of superparamagnetic nanoparticles in the size range of 10–15 nm.

Unfortunately, such particles are not favorable for aimed hyperthermia heating applications, since for maximum hyperthermia heating performance, particles with a slight ferrimagnetism are needed [11,12]. An increase in particles size leads to a transition to ferrimagnetism [13] but also an increased tendency to form agglomerates. A compromise is the application of so-called multicore magnetic nanoparticles (MCNP) with superferrimagnetic behavior, which means ferrimagnetic behavior in the presence of an external magnetic field and superparamagnetic behavior in its absence, which results in a good stability against agglomeration. These particles are very promising for medical applications [14], especially for magnetic fluid hyperthermia because of their high heating performance [15,16]. Nevertheless, these cores are in the size range of several 10 nm and exceed the here biologically favored size from 10 to 15 nm.

An approach to achieve ferrimagnetism for small MNPs is the use of cobalt ferrite as magnetic core material. Unfortunately, this material shows a very pronounced hard magnetic behavior and for particles of about 10 nm, coercivities in the order of 60 kA/m occur [17]. Such coercivities are too high to obtain a therapeutically promising heating performance when using a magnetic field strength harmless for the patient. Thus, pure cobalt ferrite is not a suitable material for the preparation of MNPs in the size from 10 to 15 nm with magnetic properties promising for hyperthermia.

**Figure 1:**
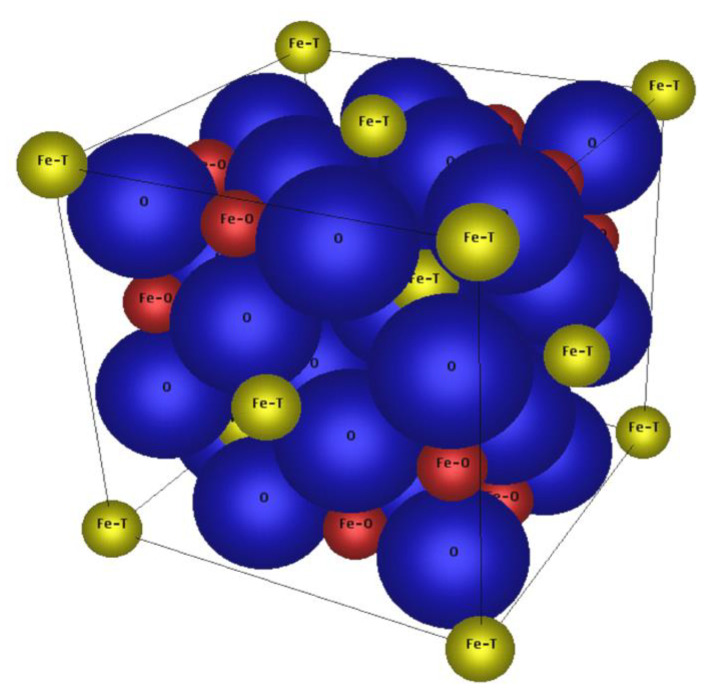
Unit cell of the spinel lattice of magnetite, consisting of 32 O^2−^ ions (O), 8 Fe^2+^ and 8 Fe^3+^ ions on the octahedral sites (Fe–O) as well as 8 Fe^3+^ ions on the tetrahedral sites (Fe–T); created with CrystalDesigner^®^.

A possible solution to prepare MNPs in the range of 10–15 nm with a mild ferrimagnetic behavior might be Co doping the magnetic iron oxides, expecting a magnetic behavior between those of superparamagnetic iron oxide and hard magnetic cobalt ferrite in dependence of the dotation degree. In the literature, a non-monotonic dependency of coercivity on Co content for non-stoichiometric compositions due to magnetically induced anisotropy is described [18]. This was also observed for nanoparticles [19] but is not expected for MNPs obtained from the here used preparation procedure. The magnetic iron oxide magnetite (Fe_3_O_4_=FeO · Fe_2_O_3_) as well as the cobalt ferrite (CoFe_2_O_4_=CoO · Fe_2_O_3_) show the crystal lattices of an inverse spinel with a cubic crystal system (Fd3m), see Figure 1. The unit cell of the inverse spinel of magnetite Fe^2+^(Fe^3+^)_2_O_4_ consists of 32 O^2−^ ions (O), eight Fe^2+^ and eight Fe^3+^ ions on the octahedral sites (Fe–O) as well as eight Fe^3+^ ions on the tetrahedral sites (Fe–T). For cobalt ferrite Co^2+^(Fe^3+^)_2_O_4_, the tetrahedral sites are occupied again by eight Fe^3+^ ions and the octahedral sites preferably by eight Co^2+^ and eight Fe^3+^ ions. This means that magnetite and cobalt ferrite have the same crystal lattice but for cobalt ferrite the eight Fe^2+^ ions on the octahedral sites are replaced by eight Co^2+^ ions, theoretically. Since the lattice constant of magnetite (*a* = 8.3985 Å) is very similar to that of cobalt ferrite (*a* = 8.3940 Å) and the ionic radii of Fe^2+^ (78 pm) and Co^2+^ (75 pm) are comparable, it should be possible to replace the Fe^2+^ step-by-step by Co^2+^ in non-stoichiometric ratios during the preparation, to tune the magnetic properties of the obtained MNP. Similar attempts were made before to improve the magnetic properties of tapes for magnetic recording [20].

For the experimental investigation of this hypothesis, magnetite was prepared by a co-precipitation process and during the preparation the Fe^2+^ content of the reagents was replaced by Co^2+^ in different ratios. The obtained MNPs were characterized structurally and magnetically. Furthermore, the biocompatibility of the MNPs after citric acid coating was investigated as well as their performance for magnetic fluid hyperthermia.

## 2. Results and Discussion

### 2.1. Structural Properties

In this study, magnetic nanoparticles (iron oxide/cobalt ferrite) with tunable magnetic properties were prepared. To adjust the magnetic properties (mainly the coercivity), during the synthesis of magnetite (Fe_3_O_4_=FeO · Fe_2_O_3_), the Fe^2+^ ions of magnetite were substituted with Co^2+^ step by step in a stoichiometric range from 0% to 33.3%. Theoretically, a substitution of 0% leads to pure magnetite, whereas 33.3% (one third of all Fe atoms are replaced by Co) leads to the formation of hard magnetic cobalt ferrite (CoFe_2_O_4_=CoO · Fe_2_O_3_) and for substitution degrees in between, Co-doped magnetite nanoparticles should be obtained. Since the preparation of the particles was performed without an exclusion of oxygen, during and after the preparation a proportion of the particle material will turn to maghemite (*γ*-Fe_2_O_3_) by further oxidation. For better understanding, the iron oxide particles will be rigorously denoted as “magnetite” in this paper.

The substitution was confirmed by means of inductively coupled plasma optical emission spectrometry (ICP–OES). For these measurements it was found that the measured Co content of the particle samples was in good accordance with the expected Co content based on the added amount of Co^2+^ ions during the preparation of the particles, see Table 1. ICP–OES determines the total proportion of elements within the particles. Thus, for a substitution of 33.3% of the Fe atoms by Co, a total amount of 25.1 wt% of Co within the MNPs was theoretically obtained. The results show that a defined amount of Co was embedded into the magnetic iron oxide particles. However, these measurements provide only information about the amount of Co within the iron oxide. A detailed investigation of the crystal lattice is needed to determine the position of the Co^2+^ within the lattice and thus, the resulting magnetic phase, see below in this section.

**Figure 2:**
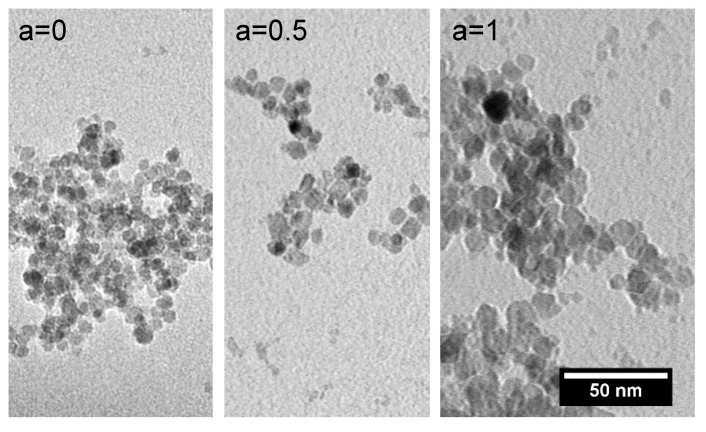
Typical TEM images of pure magnetite (**a = 0**) and the particles with a Co content of 12.5% (**a = 0.5**) as well as 25% (**a = 1**) confirm a slight increase in the particle size for increasing the Co content of the particles. The aggregation of the particles occurs during the sample preparation (drying of the fluid) for the TEM imaging.

The investigation of the dried ferrofluid samples (citric acid-coated particles dispersed in water) by means of transmission electron microscopy (TEM) revealed an overall spherical structure for the single particles, a relatively narrow particle size distribution and a low tendency to form agglomerates for all particles with different Co contents, see Figure 2. The mean particle size for all samples was in the order of 10 nm, see Table 1. For an increasing amount of Co in the particles, a slight increase in the particle core diameter was observed. For the magnetite particles (a = 0), a mean size of 8.6 nm was determined, which increased to 9.7 nm for a Co content of 12.5% (a = 0.5) until it reached 12.1 nm for a Co content of 25% (a = 1).

The investigation of the hydrodynamic diameter of the citric acid-coated magnetic cores dispersed in water by means of dynamic light scattering (DLS) revealed for all the samples a hydrodynamic diameter of 101.3 ± 6.1 nm. No correlation between the hydrodynamic diameter and Co content was observed, see Table 1. This confirmed a weak agglomeration tendency and a good stabilization of the cores against sedimentation due to the citric acid coating.

**Figure 3:**
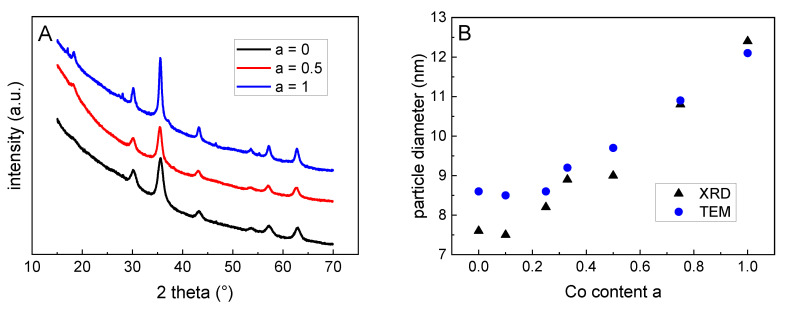
(**A**) X-ray diffractograms of the particles confirm a spinel structure of the particles; for better visibility the curves were shifted manually. (**B**) Particles size derived from XRD and TEM increase upon rising Co content.

By means of X-ray diffraction (XRD), the phase composition and the particle size was investigated. For the plain magnetite (a = 0) and for the cobalt ferrite (a = 1), the found diffraction patterns of an inverse spinel with peak patterns typical for magnetite and cobalt ferrite confirmed the formation of these magnetic phases. For all substitution degrees between 0 and 1, no remarkable changes in the diffractograms were observed, which excludes the formation of impurity phases for non-stoichiometric particles, see Figure 3A. For a = 0, the lattice constant was determined to be 8.3537 Å, which was very close to that of maghemite (8.3515 Å). In combination with a diffraction angle of 62.85° for the 440-peak of this sample, these results confirmed the magnetic phase to be maghemite for the pure iron oxide (a = 0) [21]. When a increases, the lattices constant increases up to 8.3791 Å for a = 1, which is in the order of what it is for magnetite (8.3985 Å) and cobalt ferrite (*a* = 8.3940 Å). Unfortunately, XRD did not allow us to distinguish between the magnetite and cobalt ferrite.

By the analysis of the peak broadening of the 440-peak using the Scherrer method, the mean particle sizes were derived from the scattering volume of the particles. The here obtained values confirm the results from the TEM—an increasing Co content leads to a nearly linear slight increase in the particle diameter, see Figure 3B and Table 1.

From these results it can be concluded that non-stoichiometric Fe^2+^/Fe^3+^ ratios during the particle preparation did not lead to a solid solution of different magnetic phases but rather to an inverse spinel structure with varying ratios of Fe^2+^ to Co^2+^ within the crystal lattice, probably on the octahedral gaps. This means a linear transition from magnetite to cobalt ferrite when changing the amount of Co^2+^ during the precipitation without remarkable changes in particle size. Since from the XRD data solely this hypothesis cannot be proved, a detailed investigation of the lattice structure of the particles, their magnetic alignment behavior and Fe-site occupation was performed via Mössbauer spectroscopy, see this section below.

**Figure 4:**
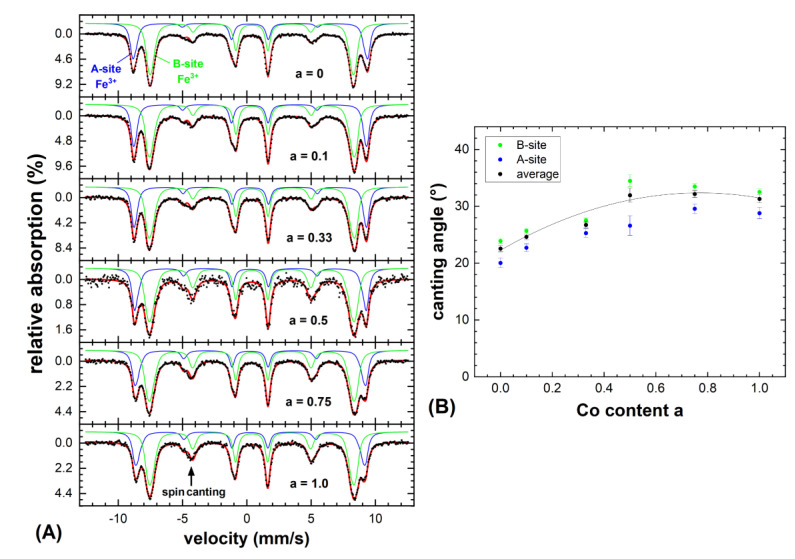
(**A**) Mössbauer spectra of the Co_a_Fe_3-a_O_4_ nanoparticles recorded at 4.3 K in a magnetic field of 5 T applied along the *γ*-ray incidence direction, composed of Fe^3+^ ions on the tetrahedral A-(blue) and the octahedral B-sites (green). Narrow hyperfine field distributions were used to reproduce the individual subspectra. (**B**) Spin canting angles extracted for the A- and B-site iron and averaged canting angles; the line serves as a guide to the eye only.

Figure 4A displays the Mössbauer spectra of the Co_a_Fe_3-a_O_4_ nanoparticles (a = 0–1) recorded at 4.3 K. To resolve the individual subspectra of Fe-ions on the tetrahedral A-(blue) and on the octahedral B-sites (green), a magnetic field of 5 T was applied along the propagation direction of the incident *γ*-ray. These two observed contributions correspond to Fe^3+^ on the mentioned lattice sites, while for Fe^2+^ a further spectral component of higher isomeric shift and lower hyperfine magnetic field would be expected, which is not visible here [22,23]. In agreement with lattice constants from the XRD, this indicates at least the partial oxidation of the relatively small Fe_3_O_4_ (a = 0) nanoparticles to maghemite (*γ*-Fe_2_O_3_), although minor contents of Fe^2+^ could be overlooked due to partial superposition with the larger B-site Fe^3+^ component. To reproduce these sextet structures, narrow distributions of effective magnetic fields were used to account for the minor variations in the local Fe-surroundings and in spin canting.

As the subspectral intensities are in good approximation proportional to the number of Fe ions on the respective lattice positions, we can obtain information on the Co site occupation by studying the ratio of A- to B-site intensity *R_AB_*. For a = 0, we obtained *R_AB_* ≈ 0.59 ± 0.03, in agreement with the assumption of partially oxidized magnetite, as for magnetite twice as much B- than A-site positions are occupied by Fe (*R_AB_* = 0.5), while in maghemite the ratio increases due to B-site vacancies (*R_AB_* = 0.6). Unlike in magnetite and maghemite, in Co-bearing samples R_AB_ will also reflect the Co-site occupation. This is often described in terms of the so-called inversion parameter S [24], which defines the occupation of the additional metal ion rather on the tetrahedral A-site in a regular spinel (*S* ≈ 0) or completely on the B-site in an inverse spinel (*S* = 1), as one would deem more likely due to the B-site preference of Co^2+^ [25]. Still, even upon increasing the Co-content, there was no significant variation in relative A- to B-site intensity, which would display a preferred B-site occupation. Instead, for a higher Co^2+^ fraction, R_AB_ slightly decreased. For a = 1.0 (CoFe_2_O_4_) we observed *R_AB_* ≈ 0.53 ± 0.05, rather indicating a random placement of Co^2+^ on both available lattice positions instead of the strongly preferred occupation of the octahedral B-site. However, in the context of this study, this can be considered as favorable, as the random placement of the Co^2+^ ions has been repeatedly reported to lead to increased saturation magnetization as compared to the inverse spinel structure due to the higher uncompensated B-site sublattice magnetization [26,27].

In addition to the site occupation probabilities, the Mössbauer spectroscopy provided valuable insight into the site-selective orientation of magnetic moments, as the average angle (canting angle *θ*) between the *γ*-ray—identical here to the direction of the applied magnetic field—and the Fe spins can be determined from the intensity ratio of lines 2 and 3 (respectively 5 to 4) of each spectral component following Fermi’s golden rule [28]. B-site canting dominates here as often observed in spinel systems, as is expected due to antiferromagnetic B–B superexchange in addition to the superior ferrimagnetic structure [29,30]. More surprisingly, Figure 4B displays an increase in *θ* upon rising Co content, with a maximum ca. at *a* = 0.6–0.8. While on the one hand smaller nanoparticles exhibit an increasingly stronger spin frustration due to their higher specific surface, on the other hand the higher magnetocrystalline anisotropy of cobalt ferrite as compared to the magnetite often results in stronger spin canting in samples comparable in size and crystallinity. Therefore, this observed trend in *θ* could be explained by more pronounced surface spin canting in smaller particles for *a* ≈ 0 due to their higher specific surface [31] and the higher magnetocrystalline anisotropy of CoFe_2_O_4_ for a approaching 1.0, with both contributions in superposition resulting in a maximum spin frustration at intermediate stoichiometries.

All in all, the Mössbauer spectroscopy indicated primarily Fe^3+^ ions in the six studied particle systems, showing a moderate spin canting and a minor B-site preference of the Co^2+^ ions.

### 2.2. Magnetic Properties

The quasistatic magnetic properties of the powders of the dried uncoated particles as well as ferrofluids of the citric acid-coated magnetic cores were determined by means of vibrating sample magnetometry (VSM).

**Figure 5:**
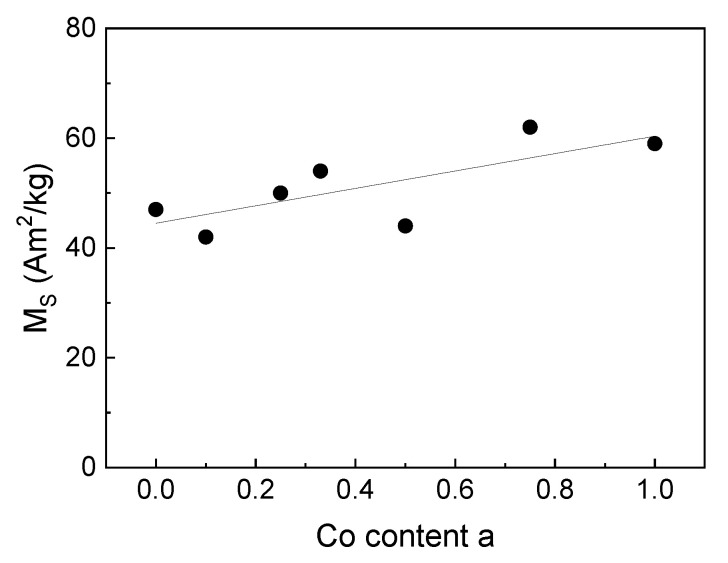
Saturation magnetization (M_S_) of powders of bare magnetic particles as function of Co content of the partices, measured by means of VSM at *H* = 1275 kA/m, the line serves as a guide to the eye only.

The saturation magnetization (M_S_) of the uncoated cores (measured at *H* = 1275 kA/m) was determined to be in the range of 42 to 62 Am^2^/kg, which represents typical values for nanoparticles of magnetite and cobalt ferrite in this size range [32]. An increasing M_S_ for increasing Co content was observed, see Figure 5 and Table 2. Since magnetite and cobalt ferrite have a similar bulk saturation magnetization of about 80 Am^2^/kg [33], the transition of magnetite to cobalt ferrite might not be the reason for the increasing M_S_ for higher Co contents. Possible reasons for this behavior might be the occurrence of maghemite (with a bulk saturation magnetization of about 60 Am^2^/kg) for lower Co content and the experimentally found increasing particles size for higher Co content, see Table 1. Thus, for the latter, for a lower Co content, smaller particles with a higher surface-to-volume ratio result. Assuming a higher ratio of non-magnetic dead layer on the surface of the particles to the total particles volume for smaller particles, smaller particles show a lower net saturation magnetization. While Mössbauer spectroscopy indicates higher spin frustration for intermediate to high Co content, these findings cannot be directly compared due to different magnetic field amplitude they were obtained at.

**Figure 6:**
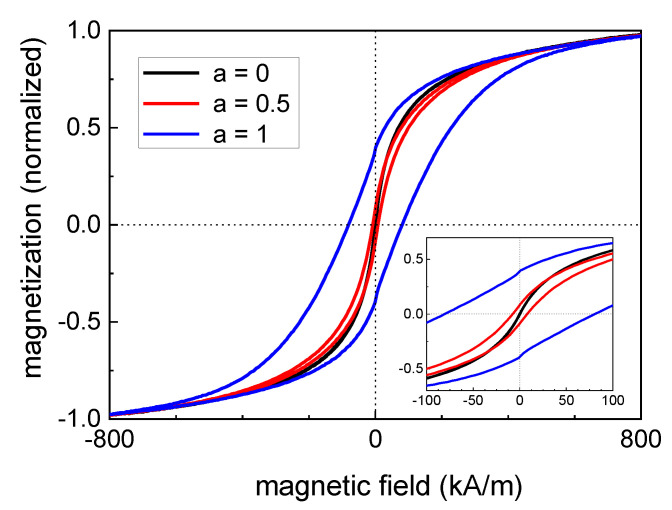
Normalized minor loops of pure magnetite (**a = 0**) and particles with a Co content of 12.5% (**a = 0.5**) as well as 25% (**a = 1**) recorded at room temperature confirm a significant increase in the coercivity for increasing Co content of the particles. The inset depicts a higher magnification of the hysteresis curves at low magnetic field strengths.

**Figure 7:**
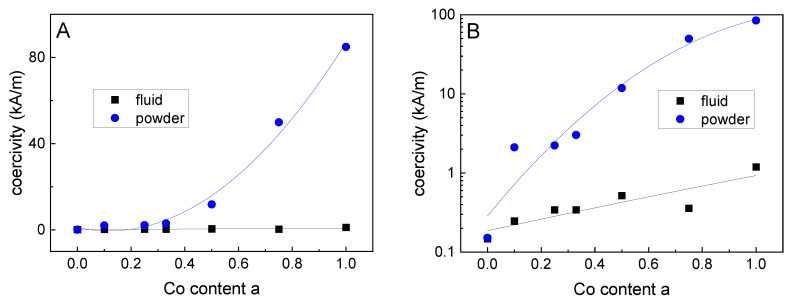
Coercivity of the powder samples (blue spheres) and the fluidic samples (black squares) as a function of the Co content, plotted on (**A**) linear and (**B**) logarithmic scales; the lines serve as a guide to the eye only.

Figure 6 shows minor loops of hysteresis curves of pure magnetite (a = 0) and particles with a Co content of 12.5% (a = 0.5) as well as 25% (a = 1), normalized to the saturation magnetization of each curve. It becomes obvious that an increasing Co content led to a more pronounced ferrimagnetic behavior (starting from a superparamagnetic behavior for the pure magnetite particles) and the coercivity H_C_ varied in a wide range. From Figure 7A,B it can be seen, that for the powder samples, a continuous increase in H_C_ took place for the increasing Co content of the particles. This means that H_C_ can be tuned in a wide range for this magnetic nanoparticle system by varying the Co content of the particles, see Table 2. This is especially true, as seen in Figure 7B, as where H_C_ is plotted on a logarithmic scale.It can be seen that even a very small amount (2.5%) of Co within the particles leads to a significant increase in H_C_ and a resulting blocked magnetic behavior at room temperature.

For the VSM measurements on liquid ferrofluid samples it was found that H_C_ shows only a very weak increase for increasing Co content, see the black squares in Figure 7A,B. This confirms the good colloidal stability of the fluids without particle agglomerates, as already found by DLS. The slight increase in H_C_ for the fluidic samples with increasing Co content might be attributed to the increasing particles size for higher Co contents. This size increase led to longer Brown relaxation times [34], resulting in a higher H_C_ for the quasistatic measurements of hysteresis curves by means of VSM. The relative remanence M_R_/M_S_ showed a dependency on the Co content similar to that found for the H_C_ (see Table 2), as described already before for other particles systems in the transition range from superparamagnetic to ferrimagnetic behavior [13].

The magnetic heating performance of the particles for hyperthermia application was investigated by a magnetic field calorimeter for field strengths of 10, 20 and 30 kA/m for a field frequency of 235 kHz. Since in animal investigations it was found that the particles were immobilized to the tumor tissue immediately after injection [15], measurements were performed on particles immobilized in gelatine.

**Figure 8:**
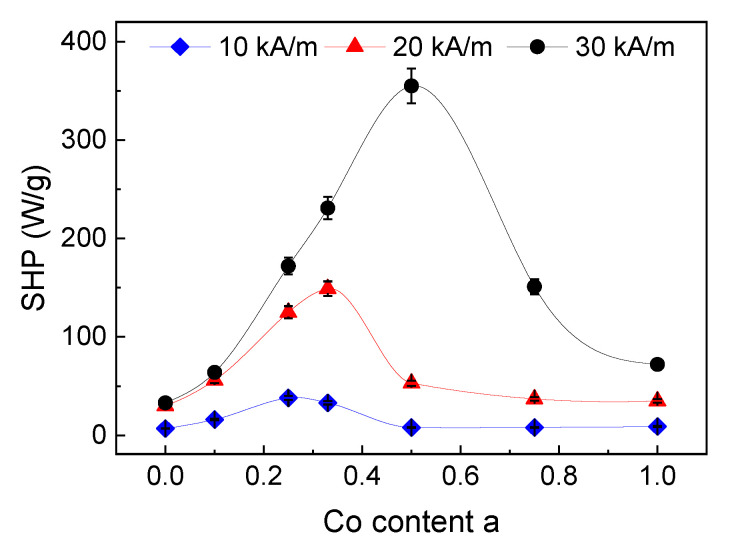
SHP of the immobilized particles for three different field strengths (10, 20, and 30 kA/m); the lines serve as a guide to the eye only, for some data points error bars are within the symbols.

In good accordance with the theory [35,36,37], the specific heating power (SHP) increased with increasing field strength in our measurements, see Figure 8. For a lower field strength (10 kA/m), a maximum SHP of 38 W/g was obtained for the sample with a H_C_ of 2.2 kA/m (a = 0.25/Co = 6.4%). In the medium field (20 kA/m), the maximum SHP is 149 W/g for the sample with a H_C_ of 3.0 kA/m (a = 0.33 (Co = 8.6%) and for the highest investigated field strength of 30 kA/m, the maximum SHP of 355 W/g was determined for the sample with a H_C_ of 11.9 kA/m (a = 0.5/Co = 12.6%), see Table 2.

This behavior can be explained by the pairing of hysteresis losses of the magnetization curve (for a saturation magnetic field strength) and the applied field to reverse the magnetization of the particles within the alternating magnetic field generator. As shown before [13], the H_C_ can serve as a measure to estimate the hysteresis losses of a particle ensemble. For a certain magnetic field strength, the reversal losses increase with increasing H_C_ due to a higher area of the hysteresis curve until the losses reach a maximum. Exceeding this maximum, a higher H_C_ led to lower losses, since the magnetic field strength was too low to reverse the magnetization of the particle ensemble completely. Due to a (possible) particle size distribution, only a fraction of the particles ensemble will show reversal losses [11,12] and the effective net losses decrease for a further increasing H_C_. This means, for each applied field strength during hyperthermia, particles with optimum losses (H_C_) have to be chosen to obtain maximum heating.

For the here presented particle system, the heating performance of the immobilized particles was in a range which was suitable for magnetic hyperthermia treatment. The magnetic heating performance of the particles could be optimized/adapted for the used magnetic field parameters by controlling the Co content (and thus the H_C_) of the particles. This means that the heating performance of the particles can be tuned without a considerable change in particle size.

### 2.3. Cell Viability Analysis

**Figure 9:**
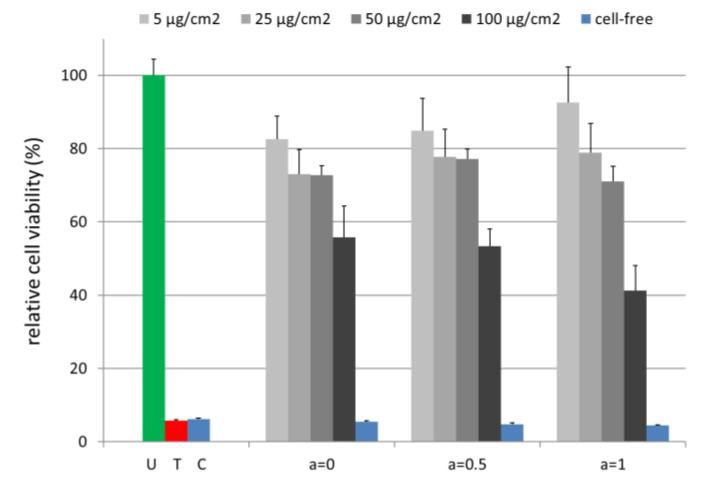
Magnetic nanoparticles (MNPs) with varying Co contents have a minor effect on the cell viability up to a concentration of 50 µg/cm^2^. The PrestoBlue Cell Viability Reagent was added after a 24 h incubation of the HBMEC samples. Cell-free samples contain 100 µg/cm^2^ MNPs. The 0.1% (*v*/*v*) Triton X 100 serves as a toxic control (T). U: untreated HBMEC control; C: cell-free medium control. *n* = 4–8 technical replicates.

Cell viability analysis enables a statement on the potential cytotoxic effects of the tested MNPs. Human brain microvascular endothelial cells (HBMECs) are a prevalent cell line representing the human blood–brain barrier [38]. In previous studies we investigated their interactions with MNPs where the cells showed a pronounced sensitivity for e.g., polyethylene imine-coated magnetic nanoparticles [39,40]. To study the effect of cobalt ferrite MNPs on human cells, HBMEC cultures were incubated with MNPs in a range of 5 µg/cm^2^ –100 µg/cm^2^ for 24 h (Figure 9). Cell viability testing was performed with the PrestoBlue reagent, which was demonstrated to allow a sensitive and robust analysis [39,41]. The fluorescence intensity of the reaction product resorufin of the MNP-treated samples was related to the untreated control (U). All MNPs independent of cobalt ion content exhibited a moderate concentration-dependent effect on HBMECs with at least 71.0% ± 4.1% cell viability up to a concentration of 50 µg/cm^2^. However, the highest MNP concentration (100 µg/cm^2^ = 388.8 µg/mL) severely affected the cells and reduced viability to 41.2% ± 6.9% for a = 1.

A reason for this observation might be the interference of the MNPs with the PrestoBlue reagent-based assay. It is reported for several cell viability assays that they might interfere with the investigated nanomaterials themselves, with assay components or with reaction products [39,41,42,43,44]. Therefore, we carried along cell-free controls supplemented with 100 µg/cm^2^ of MNPs. These samples did not exhibit enhanced fluorescence signals compared to the cell-free controls without MNPs (cell-free medium control = C) (Figure 9). To prove whether MNPs interfered with the PrestoBlue reagent product resorufin we measured the cell viability of the untreated cell cultures before and after supplementation with 100 µg/cm^2^ of MNPs. These analyses showed a marked decrease in the fluorescence intensity after the addition of MNPs. This pointed clearly to an interaction of the fluorescent reaction product resorufin with the MNPs and thus a masking of the fluorescence signal (Figure S1). In consequence, the cell viability may be underestimated at least for a MNP concentration of 100 µg/cm^2^ and especially for a = 1.

Similar drawbacks are reported for the 3-(4,5-Dimethylthiazol-2-yl)-2,5-diphenyltetrazolium bromide (MTT) assay which is frequently used to determine the cytotoxicity of nanomaterials, e.g., cell lines release the MTT formazane by exocytosis under nanoparticle incubation [45] or some nanoparticles absorb fluorescence like carbon black [46]. In general, cell viability evaluation in vitro depends on various parameters. The characteristics of the nanomaterial examined including its shape, composition and charge have to be considered as well as the concentration applied and the duration of the treatment. Furthermore, the in vitro test system like the cell line used or the incubation medium composition has to be taken into account. Moreover, the assay characteristics like the ingredients and the measuring principle play a role [47]. Therefore, it is advisable to evaluate the effects of nanomaterials on cell viability with independent assay systems.

It has to be emphasized that for the medical application of MNPs, typically a concentration of up to 25 µg/cm^2^ (97.2 µg/mL) was used. In this range, the herein tested MNPs showed only mild cytotoxic effects. Comparing for each single concentration the cell viability for different a-values, it can be seen that a higher Co content did not lead to increasing cytotoxic effects for concentrations up to 50 µg/cm^2^. Only when the concentration exceeded 50 µg/cm^2^ a higher Co content lead to stronger toxic effects. However, for such concentrations, even the pure iron oxide (a = 0) shows cytotoxicity.

In order to verify the observed effects of MNPs on cell viability with an independent approach, real-time cell analysis (RTCA) was performed. This label-free and impedance-based technique monitors the biological condition of cells, e.g., the cell number, viability and the adhesion degree [48]. RTCA avoids the interference of MNPs with dyes and allows the monitoring of the dynamic responses of the cells in real-time [49,50].

**Figure 10:**
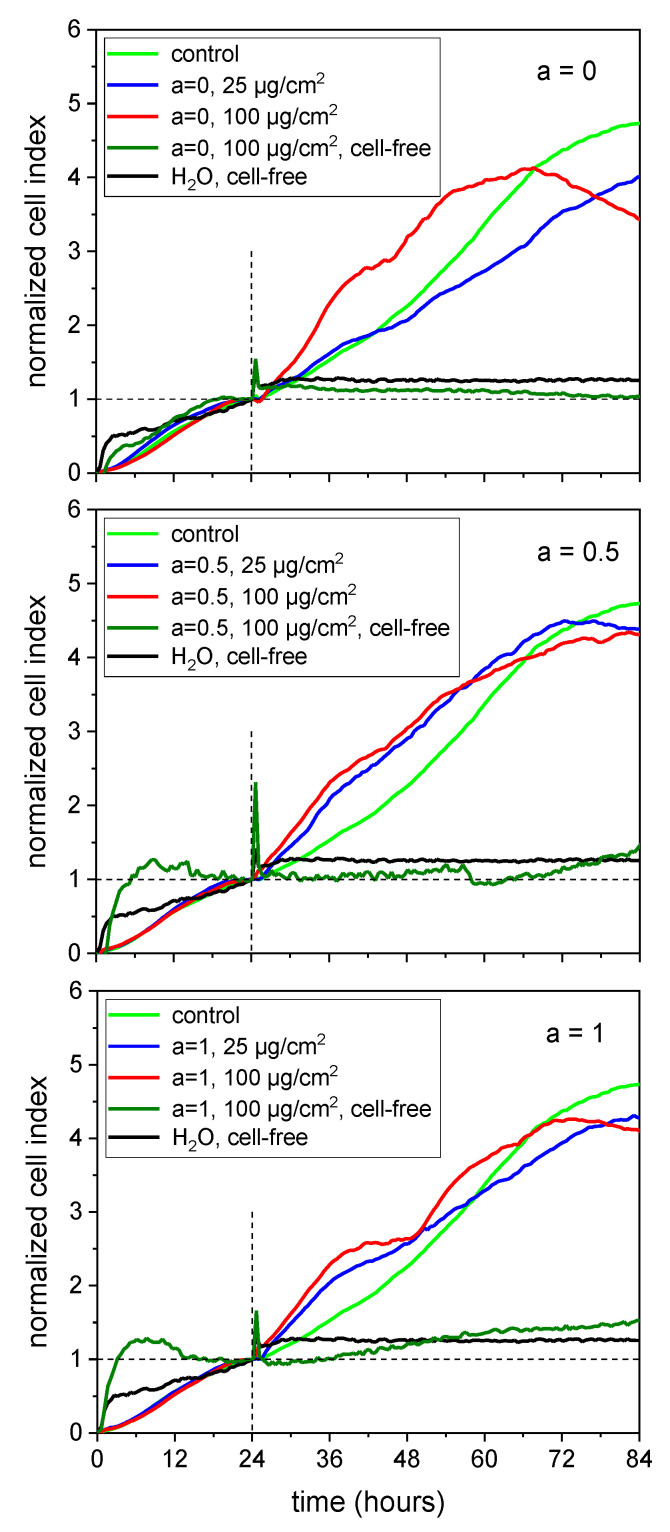
Real-time cell analysis confirmed no reduced viability of human brain microvascular endothelial cells (HBMECs) within 60 h for particles with an increasing Co content in comparison to the untreated control (green line); MNPs were added after 24 h which were indicated by the vertical dashed line. The basis of the normalized cell index was the 24 h value of the samples, respectively (horizontal dashed line). *n* = 2 technical replicates.

In our experiments, the MNPs were added 24 h after seeding the HBMECs into E16 plates. The development of the cell culture under treatment was monitored up to 84 h which means an observation period of 60 h. Figure 10 shows the results of RTCA for a = 0 (**A**), a = 0.5 (**B**) and a = 1 (**C**). The data were normalized to the time point of the MNP addition (24 h) and denoted as the normalized cell index. The untreated control showed a steady increase during the observation period. The addition of 25 µg/cm^2^ of the cobalt ferrite MNPs did not affect the progress of the cell culture. Nevertheless, it has to be mentioned that the normalized cell index was below the untreated control. Cell cultures incubated with 100 µg/cm^2^ MNPs showed a tendency towards a reduction of the normalized cell index after 60 h of treatment. The MNPs per se had no effect on the measurement over the whole period of time (see cell-free samples). Thus, the RTCA supported the idea that cobalt ferrite MNPs do not sustainably affect cell viability up to a concentration of 50 µg/cm^2^.

**Figure 11:**
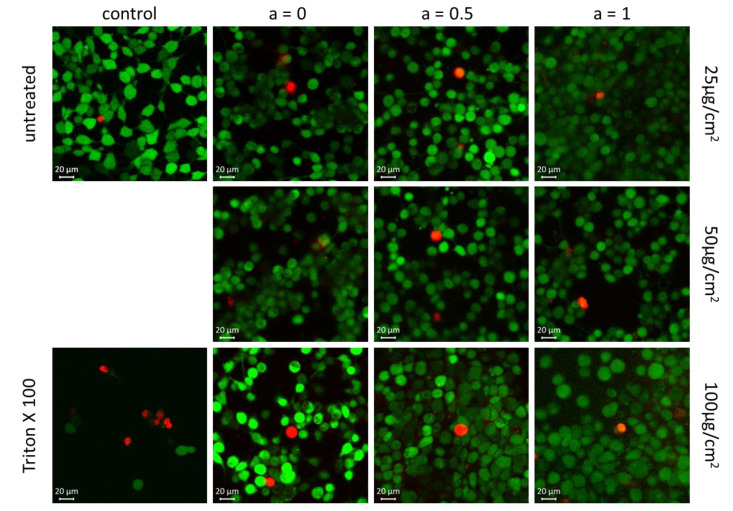
Vital fluorescent staining confirmed no reduced cell viability within 24 h for particles with an increasing Co content; the living cells and dead cells are colored in green and red, respectively. Magnification × 400.

The results obtained from the PrestoBlue assay and RTCA were confirmed by vital fluorescent staining microscopy. For that, the HBMECs were incubated with 25, 50 and 100 µg/cm^2^ MNPs for 24 h. In vital cells, the non-fluorescent calcein AM is converted into green-fluorescent calcein by intracellular esterases. Ethidium-homodimer-1 is only able to enter cells when the cell membrane is corrupted, thus labelling dead or damaged cells which are prone to die. As demonstrated in Figure 11, neither iron oxide MNPs (a = 0) nor Co-doped MNPs (a = 0.5 and a = 1) affect the viability of HBMEC. Only a few dead cells are detectable. The ratio of these cells is comparable to the untreated control. Thus, no elevated toxicity can be observed when the iron oxide MNPs are doped with cobalt ions.

Co–ferrite MNPs were investigated from several groups for their potential cytotoxicity with diverse results [47]. Horev-Azaria and colleagues could demonstrate using several cell lines that Co–ferrite MNPs exhibited a concentration-, cell-line- and duration-dependent cytotoxic effect [51]. They calculated an overall threshold of 200 µM for all cell lines, which was about 13 µg/cm^2^ in our system. Ansari et al. used a modified MTT assay and reported that nanosized cobalt ferrites exhibited only a minor toxic effect in a breast cancer cell line up to a concentration of 300 µg/mL [52].

Resulting from the cell viability experiments with citric acid-coated MNPs it can be concluded, that for concentrations suitable for medical applications, no increased toxicity in our in vitro model was observed for increasing the Co content of the MNPs. The cobalt ferrite particles show almost the same non-toxic behavior as the iron oxide particles, which are approved for medical application.

## 3. Materials and Methods

### 3.1. Particle and Ferrofluid Preparation

The magnetic nanoparticles were prepared by co-precipitation in an alkaline media applying a modified protocol of the standard preparation procedure for such particles [53]. For tuning the magnetic properties, the Fe^2+^ ions of magnetite were substituted by Co^2+^ step by step in our study which resulted in a Co-doped inverse spinel lattice with an adjustable Fe^2+^ substitution degree, see Formula (1):

(1) $${\left(Co^{2+}\right)}_{a}+{\left(Fe^{2+}\right)}_{1-a}+{\left(Fe^{3+}\right)}_{2}+{\left(OH^{-}\right)}_{8}\to \;Co_{a}^{2+}Fe_{1-a}^{2+}Fe_{2}^{3+}O_{4}+4H_{2}O\;\mathrm{with}\;a=0\ldots 1$$

**Formula (1).** Reaction equation for the preparation of Co-doped inverse spinels.

A stoichiometric substitution of Fe^2+^ by Co^2+^ of 0% (a = 0) leads to the formation of pure magnetite particles and at a substitution degree of 33.3% (a = 1), one third of all Fe^2+^ ions are replaced by Co^2+^ and pure CoFe_2_O_4_ results.

The particles were prepared from Co^2+^, Fe^2+^, and Fe^3+^ chloride mixtures (0.02 molar) at different “a” values by co-precipitation with sodium hydroxide under stirring at 100 °C for a 90 min reaction time. The obtained particles were washed with distilled water using a magnetic separation technique and stabilized in water with hydrochloric acid (intermediate with positively charged particles). Subsequently, the particles were coated with citric acid, which resulted in negatively charged MNPs at pH7. Finally, the citric acid-coated MNPs were filtrated by a 0.8 µm filter to remove potentially existing agglomerates.

### 3.2. Structural Characterization

**ICP**–**OES**: The elemental composition of the prepared nanoparticles was determined by means of inductively coupled plasma optical emission spectrometry (ICP–OES; Agilent 725 ICP–OES Spectrometer, Agilent Technologies). For the ICP–OES measurement, the nanoparticles were precipitated and dissolved in aqua regia.

**TEM**: For TEM from aqueous solutions, copper grids were rendered hydrophilic by argon plasma cleaning for 30 s. Then, 10 μL of the respective sample solution was applied to the grid, and excess sample was blotted with a filter paper. TEM images were acquired with a JEM 2010FEF (JEOL, Japan).

**XRD**: The inner structure of the magnetic nanoparticles was investigated by means of X-ray diffraction (XRD, Panalytical X’pert Pro, Malvern Panalytical, Almelo/The Netherlands). The results of the XRD investigations provided information about the magnetic phase composition and the mean size of the magnetic cores. The size of the cores was calculated from measurements of the XRD line width by using the Scherrer formula. The lattice constant was determined by means of Rietveld analysis.

**DLS**: The hydrodynamic diameters (dh) of the coated cores were determined by using dynamic light scattering (DLS, Zetasizer nano ZS, Malvern Instruments, Malvern, UK). Before the measurement, samples were diluted in the ratio 1:30 with distilled water and treated in an ultrasonic bath. For size measurements, the z-average of the intensity weighted normalization was used. All measurements were performed in 3 consecutive runs and the obtained values were averaged.

**Mössbauer spectroscopy**: Mössbauer spectra of the nanoparticle samples were recorded in transmission geometry and constant acceleration mode with a ^57^Co(Rh) source. Spectra at 4.3 K were measured in a custom-built Thor Cryogenics l-He cryostat containing a setup in split-coil geometry, providing a field of 5 T along the incidence direction of the γ-ray. Individual subspectra were reproduced by using a narrow distribution of effective magnetic fields for the A- and B-sites using the “Pi” program’s Mössbauer fitting routine [54].

### 3.3. Magnetic Characterization

**VSM**: The magnetic properties were measured at room temperature by using vibrating sample magnetometry (VSM; Micromag 3900, Princeton Measurement Systems, USA). The measurements were performed on liquid samples or dried powders. The overall magnetic behavior of the samples was derived from magnetization, coercivity and relative remanence measured at H = 1275 kA/m. The concentration of MNPs in the fluid was calculated from the obtained saturation magnetization of the fluid samples.

**SHP**: The specific heating power (SHP) was measured by means of magnetic field calorimetry at field amplitudes of 10, 20 and 30 kA/m and at a frequency of 235 kHz as described before [55]. The SHP of the immobilized particles was determined for particles dispersed in a gelatin gel. It was shown in previous investigations that this method yields a strong immobilization of the particles. For the measurement of the heating curves, the particle suspensions (1 mL in a 2 mL Eppendorf tube) were thermally isolated in a PUR foam block and the temperature measurements were performed by a fiber optic device (Fotemp, OPTOcon, Dresden, Germany). The MNP concentration of the samples for SHP measurements was determined by measuring the specific saturation magnetization using the VSM and assuming the specific magnetization values which were obtained from the powder samples.

### 3.4. Cell Viability Analysis

**Cell culture:** The biocompatibility of the prepared samples was studied utilizing human brain microvascular endothelial cells (HBMECs). The HBMECs were cultivated at 37 °C and 5% CO_2_ in RPMI 1640 + GlutaMAX™ I (Invitrogen, Karlsruhe, Germany) supplied with 10% (*v*/*v*) fetal bovine serum (FBS; Biochrom, Berlin, Germany), 100 U/mL penicillin and 0.1 mg/mL streptomycin (Life Technologies, Carlsbad, CA, USA).

**PrestoBlue Assay**: The cell viability was investigated by using the PrestoBlue Cell Viability Reagent (Invitrogen, Karlsruhe, Germany). The assay was based on the reduction of a non-fluorescent resazurin-based reagent to fluorescent resorufin by metabolically active cells. First, the cells were seeded into black-walled 96-well plates (*µ*-Clear, *F*-bottom, Greiner Bio-One, Frickenhausen, Germany) and cultivated for 24 h. The subconfluent cell cultures were incubated with particle concentrations between 5 and 100 µg/cm^2^ (which equaled 19.4 to 388.9 µg (MNPs)/mL) in at least quadruplicate. These values had a mean total Co concentration from 2.4 to 48.6 µg/mL in the case of a = 0.5 and from 4.9 to 97.2 µg/mL in the case of a = 1.0. For the control, sterile water was added (untreated control) or the detergent Triton X 100 (0.1%(*v*/*v*)). After 24 h of nanoparticle incubation the PrestoBlue reagent was added and the incubation was continued for a further 30 min at 37 °C. After magnetic separation of the MNP towards the outer walls of the wells and an excitation of the cells with light of 550 nm (9 nm bandwidth), a fluorescence signal at 600 nm (20 nm bandwidth) was detected by using the microplate reader Infinite M200 Pro (Tecan, Crailsheim, Germany). By measuring cell-free wells with added particles, the particle-associated auto fluorescence effect was measured and used for the correction of the cell measurements. Similarly, quenching effects were considered by fluorescence measurements immediately before and after the addition of particles to the cells in the wells. From these investigations, the cell survival rate (which was an indicator for the cell toxicity of the particles) was derived for the different particle concentrations at 24 h after the start of particle–cell incubation.

**RTCA**: The long-term viability of incubated HBMECs was investigated by means of real-time cell analysis (RTCA) using the xCELLigence system by ACEA Biosciences (San Diego, CA, USA) as described previously [40]. For this, 10,000 cells/cm^2^ were seeded in each well of a 16 well E plate. The cells were monitored in real time at 37 °C in a humidified atmosphere with 5% CO_2_ after a sedimentation waiting step of 30 min. After 24 h cultivation, the particles (25 µg/cm^2^ and 100 µg/cm^2^) were added to the cells and mixed carefully. RTCA measurements were continued 60 h after adding the MNPs. Plain incubation media served as untreated controls.

**Vital fluorescent staining microscopy**: The influence of the here prepared MNPs on the viability of the cells was examined by determining the ratio of living cells to dead cells using LIVE/DEAD viability/cytotoxicity fluorescent staining microscopy as described previously [40]. The HBMECs were seeded on glass cover slips (12 mm diameter, Menzel, Braunschweig, Germany) in 24-well tissue culture plates (76,000 cells/cm^2^, Greiner Bio-One). After overnight cultivation, the cell cultures were incubated with 25, 50 or 100 µg/cm^2^ of the MNP for 24 h, respectively. The cells were washed with PBS and 100 µL of an ethidium homodimer 1/calcein AM mix (0.3 µM calcein AM and 0.7 µM ethidium homodimer 1 in PBS) (Thermo Scientific, Waltham, MA, USA) was added for 3 h. Calcein (excitation: 494 nm, emission: 517 nm) and ethidium homodimer-1 (excitation: 528 nm, emission 617 nm) fluorescence was measured using the confocal laser scanning microscope LSM 510 META (Carl Zeiss Microscopy GmbH, Jena, Germany).

## 4. Conclusions

In the here presented study, magnetic Co-doped iron oxide nanoparticles with tunable magnetic properties were prepared. For the Co-doping, Fe^2+^ ions were replaced by Co^2+^ in the educts in non-stoichiometric ratios during the preparation, leading to the formation of pure iron oxide, pure cobalt ferrite and non-stoichiometric intermediates between both. The Co content of the obtained particles was in good accordance with the Co amount in the educts. The determination of the core size of the particles revealed a mean particle size in the range of 8–12 nm with the slight tendency of increasing particle sizes for higher Co contents. The measurement of the hydrodynamic diameter of the particles after the citric acid coating showed nearly the same behavior for all the investigated particles (D_h_ ≈ 100 nm), which confirmed that the particles showed a low agglomeration tendency which was not influenced by the Co content. The Mössbauer spectroscopy revealed predominantly Fe^3+^ in all the samples, while also verifying an ordered magnetic structure with low to moderate surface spin canting. Relative spectral areas of the Mössbauer subspectra indicated a mainly random distribution of Co^2+^ ions rather than the more pronounced octahedral site-preference of bulk cobalt ferrite. The magnetic properties of the obtained particles at room temperature are determined by the Co content. An increasing Co content leads to a linear increase in saturation magnetization in the range of 42–62 Am^2^/kg. The coercivity and relative remanence increased in a non-linear manner with increasing Co content. The higher coercivity for the particles with a higher Co content cannot be explained by the larger diameter of those particles but rather by the increasing crystal anisotropy due to the doping. A clear correlation between the coercivity and the magnetic heating performance for the different magnetic field strengths was found. The SHP values for the most promising particle–field combination seem suitable for application in magnetic fluid hyperthermia. By means of cell viability analysis utilizing different independent assays and procedures, no cytotoxic effects were observed for all prepared coated MNPs (all different Co content cores with citric acid coating) up to 50 µg/cm^2^. It is noteworthy that no increased toxicity was found for the Co-doped particles compared to the pure iron oxide ones, which are approved for medical applications.

In summary, we prepared biocompatible ferrofluids consisting of magnetic nanoparticles suitable for application in magnetic hyperthermia. The magnetic properties can be tuned by doping with Co in a wide range without a significant influence on the particles size. This enables an adaption of the magnetic properties of the used particles to the field parameters of the hyperthermia setup, resulting in the optimized heating performance without any change in particles size. In ongoing work, we focus on the investigation of the doping range where the transition from superparamagnetism to the blocked ferrimagnetic nanoparticle state occurs.

## Figures and Tables

**Table 1:** Overview on the structural properties of the prepared particles: the Co substitution degree (**a**) during preparation, the expected Co content (theoretically) as well as the measured resulting Co content (inductively coupled plasma optical emission spectrometry (ICP–OES)), the diameter from TEM (TEM) and XRD (XRD) as well as the hydrodynamic diameter (D_h_) from dynamic light scattering (DLS); SD = standard deviation.

a	Co Content			D					D_h_	
	Theroretic	ICP-OES		TEM	SD		XRD		DLS	SD
	[%]	[%]		[nm]	[nm]		[nm]		[nm]	[nm]
0.00	0.00	0.0		8.6	±2.1		7.6		106.2	±2.1
0.10	2.51	2.5		8.5	±2.4		7.5		94.6	±1.8
0.25	6.28	6.4		8.6	±2.0		8.2		102.1	±2.2
0.33	8.29	8.6		9.2	±2.5		8.9		97.8	±1.9
0.50	12.56	12.6		9.7	±2.8		9.0		93.4	±1.6
0.75	18.83	19.3		10.9	±2.9		10.8		106.5	±2.1
1.00	25.11	25.1		12.1	±3.1		12.4		108.3	±2.2

**Table 2:** Overview on the magnetic properties of the prepared particles with varying Co content: the saturation magnetization (M_S_), the coercivity (H_C_), the relative remanence (M_R_/M_S_) as well as the specific heating power (SHP) for different magnetic fields strengths during hyperthermia experiments at 235 kHz on particles immobilized in gelatine.

							SHP	
a	Co	M_S_	H_C_	M_R_/M_S_		10 kA/m	20 kA/m	30 kA/m
	[%]	[Am^2^/kg]	[kA/m]			[W/g]	[W/g]	[W/g]
0.00	0.0	47	0.2	0.00		7	30	33
0.10	2.5	42	2.1	0.03		16	56	64
0.25	6.4	50	2.2	0.03		38	125	172
0.33	8.6	54	3.0	0.04		33	149	231
0.50	12.6	44	11.9	0.10		8	53	355
0.75	19.3	62	49.9	0.27		8	37	151
1.00	25.1	59	84.9	0.42		9	35	72

## Supplementary Materials

The following are available online at https://www.mdpi.com/2079-4991/10/6/1019/s1, Figure S1: Co–ferrite MNPs interact with the fluorescent resorufin.
