# Supplementary material for: Biocompatible Magnetic Fluids of Co-Doped Iron Oxide Nanoparticles with Tunable Magnetic Properties
Source: Nanomaterials (Basel). 2020 May 27;10(6):1019. doi: 10.3390/nano10061019 (PMC7352500; doi:10.3390/nano10061019)
Supplement: Supplementary file 1 [file nanomaterials-10-01019-s001.pdf]

Supporting Information

Biocompatible Magnetic Fluids of Co-Doped Iron Oxide Nanoparticles with Tunable Magnetic Properties

Silvio Dutz [1,2,*], Norbert Buske [3], Joachim Landers [4], Christine Gräfe [5], Heiko Wende [4] and Joachim H. Clement [5]

1 Institute of Biomedical Engineering and Informatics (BMTI), Technische Universität Ilmenau, D-98693 Ilmenau, Germany
2 Department of Nano Biophotonics, Leibniz Institute of Photonic Technology (IPHT), D-07745 Jena, Germany
3 MagneticFluids, Köpenicker Landstraße 203, D-12437 Berlin, Germany; ngb.buske@gmail.com
4 Faculty of Physics and Center for Nanointegration Duisburg-Essen (CENIDE), University of Duisburg-Essen, D-47057 Duisburg, Germany; joachim.landers@uni-due.de (J.L.); heiko.wende@uni-due.de (H.W.)
5 Department Hematology and Oncology, Jena University Hospital, D-07747 Jena, Germany; christine.graefe@med.uni-jena.de (C.G.); joachim.clement@med.uni-jena.de (J.H.C.)
* Correspondence: silvio.dutz@tu-ilmenau.de; Tel.: +49-3677-691-309

Received: 29 April 2020; Accepted: 21 May 2020; Published: 27 May 2020

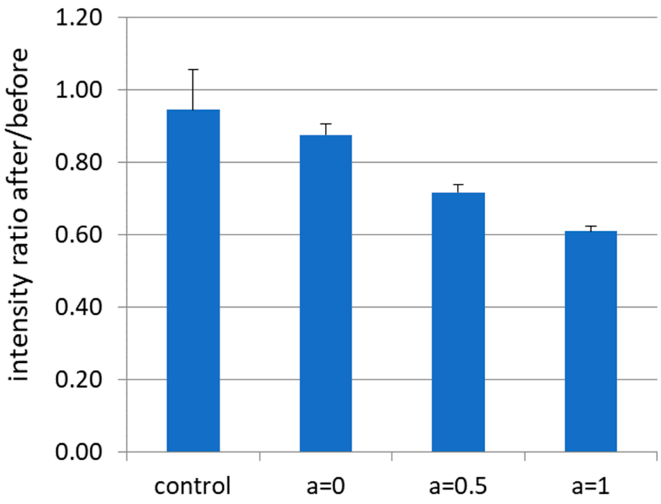

Figure S1. Co-ferrite MNPs interact with the fluorescent reaction product resorufin. Subconfluent HBMEC were treated with RPMI 1640 + 10% (v/v) FBS (equivalent to untreated control, see figure 9) for 24 hours. After a 30 min incubation with Presto Blue reagent and subsequent fluorescence measurement with the microplate reader, the cell cultures were supplemented with 100 µg/cm^2 of MNP as indicated. After magnetic separation, the samples were measured a second time. The ratio of fluorescence before and after MNP addition was determined; MNPs n = 2; control n = 8
